# Effectiveness and Cost-Effectiveness Profile of Second-Line Treatments with Nivolumab, Pembrolizumab and Atezolizumab in Patients with Advanced Non-Small Cell Lung Cancer

**DOI:** 10.3390/ph15040489

**Published:** 2022-04-18

**Authors:** Matteo Franchi, Giacomo Pellegrini, Giovanni Corrao

**Affiliations:** 1 Unit of Biostatistics, Epidemiology and Public Health, Department of Statistics and Quantitative Methods, University of Milano-Bicocca, 20126 Milan, Italy; giovanni.corrao@unimib.it; 2 National Centre for Healthcare Research and Pharmacoepidemiology, University of Milano-Bicocca, 20126 Milan, Italy; giacomo.pellegrini@unimib.it; 3 ASST Sette Laghi, Ospedale di Circolo, 21100 Varese, Italy

**Keywords:** non-small cell lung cancer, immune checkpoint inhibitors, real-world, effectiveness, cost-effectiveness

## Abstract

No evidence is available on the head-to-head comparison of clinical outcomes of patients treated with immune checkpoint inhibitors (ICIs) for advanced non-small cell lung cancer (NSCLC) in a real-world setting. We aimed to compare the effectiveness and cost-effectiveness profile of nivolumab, pembrolizumab and atezolizumab. We used a population-based retrospective cohort study based on the healthcare utilization databases of the Lombardy Region, Italy. The study cohort included all patients with a diagnosis of lung cancer, who started a second-line treatment for advanced NSCLC with nivolumab, pembrolizumab or atezolizumab from 2015 to 30 June 2020. Overall survival and average cumulative healthcare costs were measured from the start of second-line treatment until 31 December 2020. The study cohort included 1607 patients who started a second-line treatment with ICIs, of which there were 1193 with nivolumab, 138 with pembrolizumab and 276 with atezolizumab. No differences were observed between treatment arms in terms of sex, age or comorbidities. Median OS was very similar between groups, being 8.9, 9.4 and 8.7 months, respectively, in patients treated with nivolumab, pembrolizumab and atezolizumab (*p* = 0.898). The adjusted hazard ratio of death of patients treated with pembrolizumab and atezolizumab, as compared to nivolumab, were 1.01 (95% CI: 0.81 to 1.25) and 1.03 (0.88 to 1.21), respectively. Healthcare cumulative costs measured in the first two years of follow-up were EUR 43,764, 46,233 and 34,116, on average, associated with nivolumab, pembrolizumab and atezolizumab, respectively. In our real-world study, atezolizumab was the ICI associated with the most favorable cost-effectiveness profile.

## 1. Introduction

Worldwide, lung cancer represents the second most common neoplasm and the leading cause of cancer mortality [1]. Non-small cell lung cancer (NSCLC) is the most common lung cancer sub-type, accounting for about 85% of all lung cancer diagnoses [2], and about 50% of patients have advanced disease at cancer diagnosis [3]. In these patients, the 5-year overall survival (OS) is low, ranging from 26% in stage IIIB to 1% in stage IVB [4].

Immune checkpoint inhibitors, namely nivolumab, pembrolizumab and atezolizumab, were developed and are currently recommended as a treatment option for previously treated patients with tumor PDL1 expression [5]. In particular, current Italian treatment guidelines recommend testing for PD-L1 biomarkers in order to identify patients who are eligible for immune checkpoint therapies [6]. Although the efficacy of these treatments has been shown in randomized clinical trials (RCTs) [7,8,9], very few studies directly compared clinical outcomes of patients treated with currently available immune checkpoint therapies. A network meta-analysis of RCTs indirectly comparing the efficacy of second-line treatment with immune checkpoint treatments [10], and real-world studies comparing nivolumab with atezolizumab in previously treated patients [11,12], found no significant differences in clinical outcomes among these therapies.

In order to fill this gap, we assessed the real-world comparative effectiveness of nivolumab, pembrolizumab and atezolizumab as second-line treatment in a large and unselected population-based cohort of advanced NSCLC patients in the Lombardy Region, Italy. In addition, we evaluated healthcare costs and the cost-effectiveness profile associated with these therapies.

## 2. Results

**Figure 1:**
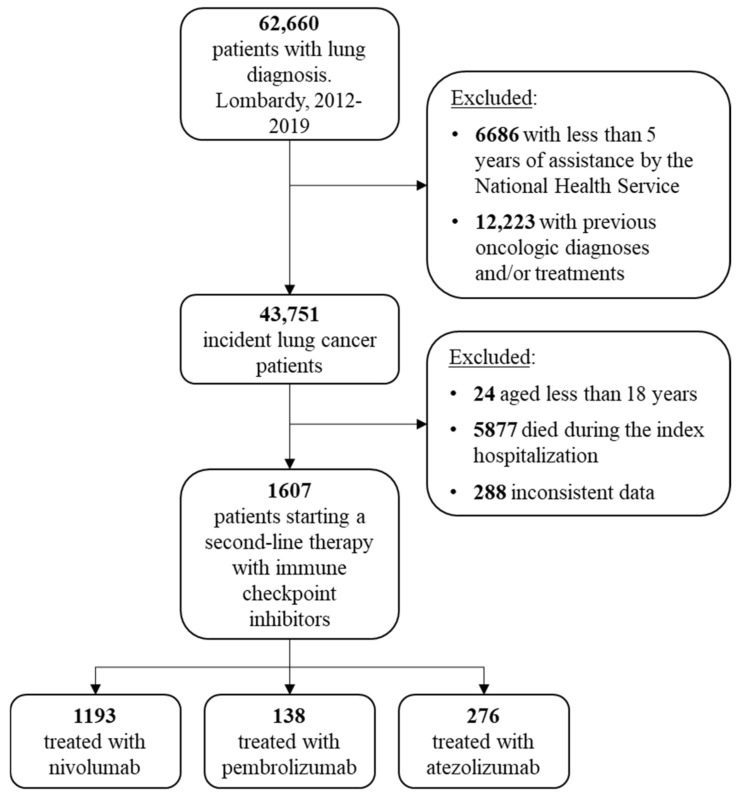
Flowchart of cohort selection.

The flowchart of cohort selection is shown in Figure 1. The study cohort included 1607 advanced NSCLC patients who started a second-line therapy with immune checkpoint inhibitors until 30 June 2020. Among these, 1193 patients started a second-line treatment with nivolumab, 138 with pembrolizumab and 276 with atezolizumab. Baseline characteristics of patients included in the study cohort are shown in Table 1.

About two-thirds of patients were male, and about 50% of patients were aged 70 years or older, with no differences between treatment arms (*p*-values were 0.318 and 0.509, respectively). The cancer multimorbidity score was distributed homogeneously between treatment arms (*p* = 0.687). A different distribution in the year of second-line treatment start was observed, with nivolumab first prescribed late in 2015, pembrolizumab in 2017 and atezolizumab in 2018, reflecting the different dates of approval of these immune checkpoint inhibitors in Italy.

### 2.1. Survival Analysis

**Figure 2:**
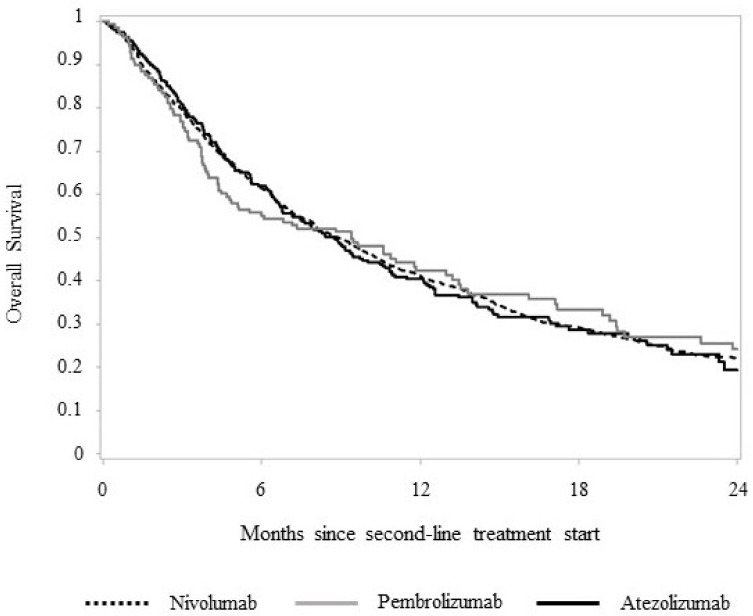
Two-year overall survival of 1607 advanced NSCLC patients starting a second-line systemic treatment with nivolumab, pembrolizumab and atezolizumab.

After a mean follow-up of 10.5 months, 899 (75.4%) deaths were observed among patients treated with nivolumab, 92 (66.7%) in patients treated with pembrolizumab and 189 (68.5%) in those treated with atezolizumab. No differences in OS were observed between treatment arms, median OS or RMST (Figure 2). The median OS was 8.9, 9.4 and 8.7 months, respectively, in patients treated with nivolumab, pembrolizumab and atezolizumab (*p* = 0.898). Corresponding figures of RMST were 11.4, 11.5 and 11.3 months, respectively (*p* = 0.980).

The multivariate analysis showed no differences in the risk of death of patients starting a second-line treatment with pembrolizumab or atezolizumab, as compared to those treated with nivolumab. Corresponding HR were 1.01 (95% CI: 0.81 to 1.25) and 1.03 (0.88 to 1.21), respectively. No association was found between sex and age categories for the risk of death. Conversely, a positive trend was observed between MCS categories and the risk of death, with HR increasing from 1.36 (1.17 to 1.59) to 1.88 (1.48 to 2.39) in patients with MCS between 10 and 19 and in those with MCS greater than 30, respectively, as compared to those with MCS less than 10 (Table 2).

### 2.2. Healthcare Costs

**Figure 3:**
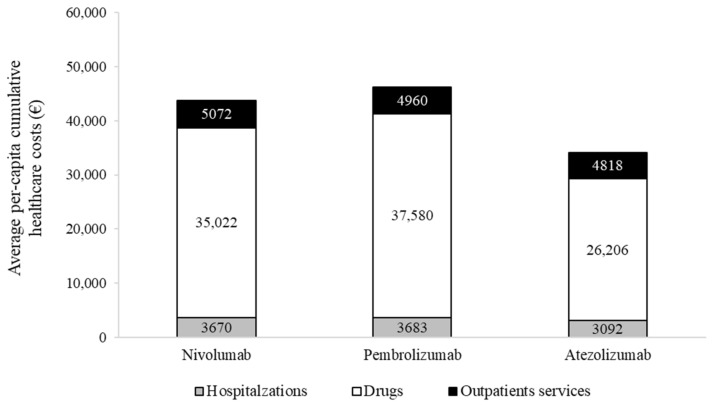
Two-year average per-patient cumulative costs, stratified by second-line systemic treatment.

Cumulative NHS healthcare costs according to second-line treatments are shown in Figure 3.

**Figure 4:**
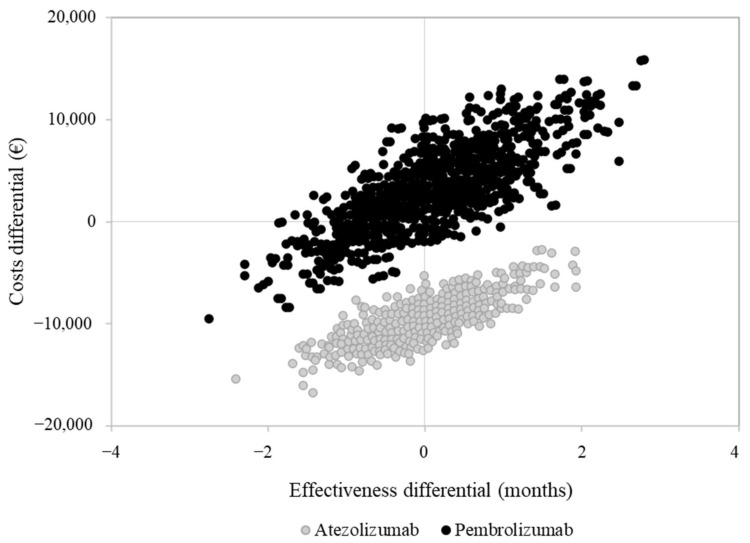
Cost-effectiveness analysis comparing second-line pembrolizumab and atezolizumab compared to nivolumab.

Overall costs were EUR 43,764, 46,233 and 34,116, on average, for a patient treated with nivolumab, pembrolizumab and atezolizumab, respectively. The average cost for a patient treated with nivolumab included EUR 3670 for hospitalization, EUR 35,022 for drugs (of which EUR 33,311 is for nivolumab) and EUR 5072 for outpatient services. Corresponding figures for a patient treated with pembrolizumab and atezolizumab were EUR 3683, 37,580 (EUR 36,270 for pembrolizumab) and 4960, and EUR 3092, 26,206 (EUR 24,965 for atezolizumab) and 4818, respectively. The cost-effectiveness profile is shown in Figure 4.

As compared to nivolumab, atezolizumab was associated with a favorable cost-effectiveness profile since patients treated with atezolizumab experienced a very similar overall survival to those treated with nivolumab, but at a lower cost. Conversely, pembrolizumab was as effective as nivolumab, but with slightly higher costs.

## 3. Discussion

In the current real-world study, we assessed the OS and healthcare costs in an unselected population-based cohort of patients with advanced NSCLC treated in second-line therapy with immune checkpoint inhibitors. We found that, although the OS did not significantly differ between treatments, a difference in cost-effectiveness profile was observed. 

Our results are consistent with those reported in other observational studies. In particular, a real-world study conducted in the United States (US) found no significant difference in OS among 2630 advanced NSCLC patients treated with second-line nivolumab, as compared to 206 patients treated with second-line atezolizumab (HR = 1.07, 0.89 to 1.28) [11]. Another US real-world investigation that compared the OS of advanced NSCLC patients treated with atezolizumab or nivolumab after progression during or after platinum-based chemotherapy found no significant difference in OS (HR = 0.77, 0.45 to 1.30) between the two groups [12]. Moreover, a network meta-analysis of RCTs, comparing the OS of advanced NSCLC with wild-type or unknown EGFR status treated according to second-line treatment, found no association between nivolumab vs. pembrolizumab (HR = 1.03, 0.77 to 1.40), nivolumab vs. atezolizumab (HR = 1.06, 0.82 to 1.37) or pembrolizumab vs. atezolizumab (HR = 1.03, 0.77 to 1.36) [10].

A recent meta-analysis of real-world studies summarized a 10-month OS among patients with advanced or metastatic NSCLC treated with second-line immune checkpoint inhibitors [13]. In our cohort, median OS was similar, being 8.9, 9.4 and 8.7 months in patients treated, respectively, with second-line nivolumab, pembrolizumab or atezolizumab.

The analysis of healthcare costs showed that, in all treatment arms, costs associated with immune checkpoint inhibitors constitute the main source of cost, accounting for about 75–80% of total costs. These results are coherent with an economic analysis performed in several European countries (including Italy), in which the estimated costs of drugs corresponded to approximately 77.4% of the total healthcare costs [14]. Among the three immune checkpoint inhibitors currently approved for treating advanced NSCLC, atezolizumab is the therapy associated with lower healthcare costs in the Lombardy setting. This data is consistent with a French cost-effectiveness study that concluded that atezolizumab is a cost-saving alternative to nivolumab, based on list price [15]. The cost-effectiveness of atezolizumab versus nivolumab for second-line treatment was also evaluated in a recent Canadian study based on RCT data, in which atezolizumab was more effective and less costly than nivolumab, although based on a modest difference in quality-adjusted life years (QALYs) and costs [16].

Our study has several strengths. First, to the best of our knowledge, this is the first study comparing head-to-head immune checkpoint inhibitors used as a second-line treatment for advanced NSCLC in terms of overall survival, healthcare costs and cost-effectiveness profile in a real-world setting. Second, given the population-based study design, our results are generalizable to the routine clinical practice in Lombardy and reflect the potential heterogeneity in the management of lung cancer patients. However, the main limitation of this study is that detailed information on the clinic and histologic biomarkers were not available in our database, so making our results vulnerable to confounding. However, a recent US real-world study comparing nivolumab vs. atezolizumab did not find significant differences in OS when considering strata of cancer stage, histology and ALK/EGFR/ROS1 variants [11]. This suggests that it is unlike that such unmeasured characteristics may affect our results. Nevertheless, we cannot exclude residual unmeasured confounding effects.

In conclusion, although OS associated with second-line nivolumab, pembrolizumab and atezolizumab was very similar, atezolizumab was the treatment associated with the most favorable cost-effectiveness profile.

## 4. Materials and Methods

### 4.1. Data Sources

We retrieved data from the health care utilization (HCU) databases of Lombardy, the most populous Italian region, with nearly 10 million individuals, covering about 17% of the entire Italian population. In Italy, the whole population is covered by the National Health Service (NHS), which provides free healthcare services for all citizens. In Lombardy, the management of NHS has been associated since 1997 with an automated system of databases to collect health information, including, among others, (i) demographic and administrative data on NHS beneficiaries, including sex, year of birth, date of death and dates of immigration or emigration; (ii) information on hospitalizations, including inpatient primary diagnosis and up to five coexisting conditions and procedures coded according to the International Classification of Diseases, 9th Revision, Clinical Modification (ICD-9-CM) classification system; (iii) drugs dispensed by territorial pharmacies and those directly administered in day-hospitals and outpatient setting, coded according to the Anatomical Therapeutic Chemical (ATC) classification system; (iv) data on outpatient services, including laboratory tests, diagnostic imaging and specialist visits. Record linkage between databases was performed by means of an identification code assigned to each NHS beneficiary. In order to preserve the privacy of the beneficiaries, identification codes were de-identified, and the conversion table was deleted.

### 4.2. Cohort Selection and Follow-Up

Details of the selection criteria of the study cohort are reported in a recent publication, in which we investigated therapeutic pathways, clinical outcomes and healthcare costs of advanced NSCLC patients treated with first-line target therapies and immune checkpoint inhibitors in the Lombardy Region [17]. Briefly, the target cohort included 37,562 patients residing in Lombardy and beneficiaries of the NHS, aged 18 years or more, with a new diagnostic code for lung cancer during the years 2012 to 2019. Among these, all patients from the target cohort who started their treatment with pembrolizumab (ATC L01XC18), nivolumab (ATC L01XC17) or atezolizumab (ATC L01XC32) within 30 June 2020 were identified. In Italy, at the time of the end of the study cohort recruitment, nivolumab and atezolizumab were only indicated as second-line treatments of advanced NSCLC after the failure of a chemotherapy-based first-line treatment, while pembrolizumab was also indicated as a first-line treatment option [6]. Thus, in order to ensure that pembrolizumab was administered as a second-line treatment, only patients who underwent chemotherapy within six months before the date of the first pembrolizumab administration were included in the final study cohort. Patients were followed-up from the date of start of the second-line treatment with either nivolumab, pembrolizumab or atezolizumab (which we named “index date”) until 31 December 2020.

### 4.3. Outcomes

The clinical outcome of interest was overall survival (OS), defined as the time between the index date and the date of death for any cause, lost to follow-up (i.e., migration to another region), 31 December 2020, or two years after index date, whichever came first.

The cost outcome was measured by the average per-capita cumulative healthcare direct costs sustained by the NHS for the treatment of patients included in the study cohort, including all inpatient and outpatient costs from index date to the earliest date between death, lost to follow-up, 31 December 2020, or two years after index date, whichever came first.

### 4.4. Baseline Characteristics

Baseline covariates included sex, age and calendar year at index date. In addition, the Cancer Multimorbidity Score (CMS), a score recently developed and validated in Italy, predictive of mortality in elderly cancer patients [18], was calculated over the two years before the index date.

### 4.5. Statistical Analyses

Descriptive tables were used for summarizing baseline characteristics. Categorical variables were described by frequencies and percentages and compared between treatment arms using the chi-squared test. OS was estimated using the Kaplan–Meier (KM) method. Median survival and restricted mean survival time (RMST) were reported as descriptive measures of survival for each treatment arm. RMST, defined as the area under the KM curve [19], represents the average survival time experienced by cohort members [20]. The association between second-line treatment and risk of death was estimated by using a multivariate Cox proportional hazard model, adjusted for sex, age and MCS categories. Estimates were expressed as Hazard Ratio (HR), along with 95% Confidence Intervals (CI).

Cumulative healthcare costs (CHC) according to second-line treatments were calculated by means of the Bang and Tsiatis estimator [21], a method that takes into account censored cost data. For each patient, CHC was calculated by summing up direct costs sustained by the NHS. The cost-effectiveness profile was assessed by dividing the between-arm differences in healthcare costs and health-related outcomes (measured by the RMST), using nivolumab as the treatment reference. The non-parametric bootstrap method based on 1000 re-samples [22] was used to explore the uncertainty in the cost-effectiveness estimates [23]. All analyses were performed using SAS 9.4 (Cary, NC, USA). Statistical significance was set at the 0.05 level. All *p*-values were two-sided.

## Figures and Tables

**Table 1:** Characteristics of 1607 advanced NSCLC patients starting a second-line treatment with nivolumab, pembrolizumab and atezolizumab.

	Second-Line Treatment			
	Nivolumab N = 1193	Pembrolizumab N = 138	Atezolizumab N = 276	*p*-Value
Sex				
Male	844 (70.7)	94 (68.2)	183 (66.3)	0.318
Female	349 (29.3)	44 (31.8)	93 (33.7)	
Age				
<60	192 (16.1)	29 (21.0)	50 (18.1)	0.509
60–69	406 (34.0)	43 (31.2)	84 (30.4)	
70–79	494 (41.4)	54 (39.1)	111 (40.2)	
≥80	101 (8.5)	12 (8.7)	31 (11.2)	
Cancer morbidity score				
<10	273 (22.9)	34 (24.6)	72 (26.1)	0.687
10–19	531 (44.5)	65 (47.1)	119 (42.1)	
20–29	304 (25.5)	31 (22.5)	61 (22.1)	
≥30	85 (7.1)	8 (5.8)	24 (8.7)	
Year of second-line treatment				
2015	2 (0.2)	0 (0)	0 (0)	<0.001
2016	140 (11.7)	0 (0)	0 (0)	
2017	428 (35.9)	29 (21.0)	0 (0)	
2018	325 (27.2)	43 (31.2)	27 (9.8)	
2019	203 (17.0)	36 (26.1)	176 (63.8)	
2020	95 (8.0)	30 (21.7)	73 (26.5)	

**Table 2:** Association between selected covariates and risk of death among 1607 advanced NSCLC patients starting a second-line systemic treatment with immune checkpoint inhibitors.

	Hazard Ratio (95% Confidence Intervals)
Second-line treatment	
Nivolumab	Reference
Pembrolizumab	1.01 (0.81–1.25)
Atezolizumab	1.03 (0.88–1.21)
Sex	
Male	Reference
Female	1.04 (0.92–1.18)
Age class	
<60 years	Reference
60–69 years	1.08 (0.91–1.28)
70–79 years	1.05 (0.89–1.25)
≥80 years	1.12 (0.88–1.42)
Cancer Multimorbidity Score	
<10	Reference
10–19	1.36 (1.17–1.59)
20–29	1.72 (1.45–2.03)
≥30	1.88 (1.48–2.39)

## Data Availability

The data that support the findings of this study are available from the Lombardy Region, but restrictions apply to the availability of these data, which were used under license for the current study, and so are not publicly available. Data are, however, available from the Lombardy Region upon reasonable request.
